# Newborn Screening of Primary Carnitine Deficiency and Prenatal Genetic Diagnosis for a Recurrence‐Risk Family

**DOI:** 10.1002/mgg3.70316

**Published:** 2026-10-11

**Authors:** Huilin Sun, Jianli Zheng, Chong Zhou, Haihong Chen, Lu Zhang, Xiaorui Li, Daocheng Bao, Fangfang Yang, Jianbing Liu

**Affiliations:** ^1^ Center for Medical Genetics Yancheng Maternal and Child Health Care Hospital Affiliated to Yangzhou University Yancheng China; ^2^ Pediatric Genetic Metabolic Endocrine Clinic Yancheng Maternal and Child Health Care Hospital Affiliated to Yangzhou University Yancheng China; ^3^ Office for Newborn Disease Screening Yancheng Maternal and Child Health Care Hospital Affiliated to Yangzhou University Yancheng China; ^4^ Department of Neonatology Yancheng Maternal and Child Health Care Hospital Affiliated to Yangzhou University Yancheng China

**Keywords:** newborn screening, primary carnitine deficiency (PCD), *SLC22A5* gene, tandem mass spectrometry

## Abstract

**Background:**

Primary carnitine deficiency (PCD) is a treatable autosomal recessive disorder caused by *SLC22A5* gene variants and included in newborn screening widely. However, its prevalence and variant spectrum vary greatly, and prenatal diagnosis for recurrence‐risk families remains rare. This study assessed tandem mass spectrometry (MS/MS) combined with genetic testing in NBS and prenatal diagnosis of PCD in Yancheng, China, and described the local prevalence and variant characteristics.

**Methods:**

From 2015 to 2024, free carnitine (C0) levels were measured in neonatal dried blood spot (DBS) samples by MS/MS for PCD screening. Newborns with persistently low C0 levels were recalled for diagnostic evaluation. At diagnosis, the C0 levels in both neonatal and maternal blood were measured, and next‐generation sequencing was performed for *SLC22A5* variants in the newborns. For the recurrence‐risk family with a previous proband, Sanger sequencing was used to confirm the parental origin of the proband's variants, and prenatal diagnosis with amniotic fluid was performed in the subsequent pregnancy.

**Results:**

Combined MS/MS and genetic testing led to the diagnosis of 17 newborns with PCD (15 molecularly confirmed with biochemical results, two clinically inferred) and three newborns with maternal carnitine deficiency among the 152 newborns with persistently low C0 levels on re‐screening. In these 17 PCD newborns, 10 distinct variant sites were detected, of which missense variants predominated, with c.1400C>G (p.S467C) being the most frequent (13/32, 40.63%). For the proband in this recurrence‐risk family, the two pathogenic variants were inherited from each parent. The fetus carried only the maternal variant, without postnatal anomalies.

**Conclusion:**

NBS using MS/MS combined with genetic testing facilitates early diagnosis and classification of PCD. The screening‐detected prevalence of PCD in Yancheng was 1:25,852, with c.1400C>G (p.S467C) as the hotspot variant. Genetic testing of *SLC22A5* provided effective prenatal diagnosis for the recurrence‐risk family.

## Graphical abstract

We performed large‐scale newborn screening for primary carnitine deficiency (PCD) using tandem mass spectrometry and genetic testing. We determined the local prevalence of PCD and the hotspot variant. Genetic testing was successfully applied for prenatal diagnosis in a recurrence‐risk family.

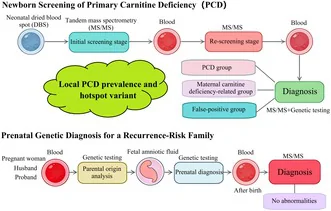

**Abbreviations**
C0: free carnitine
DBS: dried blood spot
MS/MS: tandem mass spectrometry
NBS: newborn screening
OCTN2: organic cation transporter 2
PCD: primary carnitine deficiency

## 1 Introduction

Primary carnitine deficiency (PCD, OMIM #212140) is an autosomal recessive metabolic disorder caused by variants in the *SLC22A5* gene (OMIM *603377). Approximately half of PCD patients develop hypoketotic hypoglycemia between 3 months and 2 years of age, accompanied by hepatic encephalopathy, lethargy and hyperammonemia. The remaining half develop myopathic manifestations at 2–4 years old, including skeletal muscle weakness, poor exercise tolerance, cardiomyopathy and arrhythmias. In severe cases, sudden death may occur (Dahash and Sankararaman 2023; Hanington et al. 2023; Louis et al. 2023). The global prevalence of PCD varies greatly, ranging from 1:297 in the Faroe Islands to 1:40,000 in Japan and 1:142,236 in the United States (Koleske et al. 2022; Schiergens et al. 2021). As a populous country, China exhibits distinct regional differences in PCD prevalence, ranging from 1:8938 to 1:45,000 (Ji et al. 2023).

The *SLC22A5* gene is located on chromosome 5q31.1 and encodes organic cation transporter 2 (OCTN2). OCTN2 is widely expressed in tissues including the liver, kidney, heart, skeletal muscle, and placenta, maintaining intracellular free carnitine (C0) concentrations 20 to 50 times higher than extracellular levels (Hanington et al. 2023). *SLC22A5* displays distinct variant spectra and specific hotspot variants across different ethnic groups and regions, and biallelic loss‐of‐function variants of this gene are responsible for PCD (Koleske et al. 2022; Yang et al. 2021; Zhou et al. 2024). Such variants cause OCTN2 dysfunction, leading to massive urinary loss of C0 and disrupted carnitine homeostasis, which in turn gives rise to the various clinical manifestations (Jokiel and Bermudez 2025; Kheirandish et al. 2024; Lefèvre et al. 2023).

Prior to newborn screening (NBS) implementation, diagnosis of PCD was delayed until damage occurred in multiple vital organs, bringing financial burdens and psychological stress to affected families (Gallant et al. 2017). However, PCD is a treatable inherited metabolic disorder, early diagnosis and timely L‐carnitine supplementation can improve the prognosis of PCD patients (Koleske et al. 2022; van den Heuvel et al. 2023). Currently, multiple countries and regions utilize tandem mass spectrometry (MS/MS) to measure C0 levels in neonatal dried blood spot (DBS) samples for PCD screening (Lefèvre et al. 2023; Louis et al. 2023). To enable early identification and subtyping of neonatal PCD patients, we conducted screening using MS/MS to measure C0 levels in neonatal DBS samples since 2014. This was combined with genetic testing for diagnostic evaluation of cases with positive results on re‐screening.

This study retrospectively analyzed the screening data of 152 newborns with persistently low C0 levels on re‐screening, the *SLC22A5* variants of 17 PCD newborns, and the prenatal diagnosis for one recurrence‐risk family with a previous proband. It provides references for NBS and prenatal diagnosis of PCD, as well as research on *SLC22A5* variants.

## 2 Materials and Methods

### 2.1 Study Subjects

A total of 439,486 newborns underwent PCD screening via DBS at the Newborn Disease Screening Center of our hospital from January 2015 to December 2024. MS/MS was used to measure C0 levels in DBS and blood samples. When the C0 level was < 10 μmol/L, the sample was considered positive, and the original DBS was retested. If C0 remained below 10 μmol/L, the newborn was recalled for blood collection and re‐screening. If the results were persistently positive, the newborn was recalled for diagnostic evaluation. During the diagnostic period, C0 levels in both the newborn and the mother were measured, and genetic testing was performed for the newborn.

Clinical records and relevant pathogenic variants were collected from one recurrence‐risk family with a previous PCD proband. The proband carried two heterozygous variants in the *SLC22A5* gene (NM_003060.3) (GRCh37/hg19): c.865C>T (p.R289*) and c.1400C>G (p.S467C). Both parents exhibited normal phenotypes. Peripheral blood DNA was extracted from the parents for Sanger sequencing to determine the parental origin of the proband's variants. Amniotic fluid DNA was extracted from the fetus for Sanger sequencing to verify these specific variants.

All participants or their guardians signed informed consent forms. This study was approved by the Medical Ethics Committee of our hospital (2025‐LW‐012).

### 2.2 Methods

#### 2.2.1 MS/MS

Heel blood samples were collected from newborns within 3 days of birth and spotted onto filter paper to form three blood spots with diameters > 8 mm. After natural drying, DBS samples were pretreated with the NeoBase Non‐Derivatized Tandem Mass Spectrometry Kit (PerkinElmer, USA), and C0 levels were measured using the ACQUITY TQD mass spectrometer (Waters, USA).

Sample preparation and data analysis were performed in strict accordance with laboratory standard operating procedures.

#### 2.2.2 Genetic Testing

After written informed consent was obtained, genetic testing was performed on blood samples from newborns with persistently positive results on re‐screening. The experimental operations and data analysis were completed by Zhejiang Biosan Biotechnology Co. Ltd. Genomic DNA was fragmented and used for library preparation. Libraries were enriched for the exons and flanking ±10 bp intronic regions of the *SLC22A5* gene. Sequencing was performed on the DNBSEQ‐T7 platform (MGI, China) with an average sequencing depth ≥ 200×, and 20× coverage of the target regions reached ≥ 95%. Raw sequencing reads were aligned to the human reference genome (GRCh37/hg19) using the Burrows‐Wheeler Aligner (BWA). Variant annotation was performed against the *SLC22A5* transcript NM_003060.3.

All detected variants were validated by Sanger sequencing, and pathogenicity was classified according to the American College of Medical Genetics and Genomics (ACMG) guidelines.

For Sanger sequencing, primers were designed using Primer Premier 5.0 software. PCR products were recovered and purified from agarose gels, and sequencing was performed on the ABI 3500XL genetic analyzer (Applied Biosystems, USA).

#### 2.2.3 Diagnostic Criteria

The diagnostic criteria were based on clinical practice combined with the Chinese expert consensus, “Consensus on Screening, Diagnosis, and Treatment of Primary Carnitine Deficiency” (Yang et al. 2019).

Blood C0 levels remained < 10 μmol/L in three consecutive measurements with adequate feeding during diagnostic period:

1. If two SLC22A5 gene variants were detected, the newborn was diagnosed with PCD.
2. If only one or no variant was detected, PCD could still be diagnosed after exclusion of maternal and secondary carnitine deficiency.
3. If only one or no variant was detected and the mother had blood C0 levels < 10 μmol/L, the case was diagnosed as maternal carnitine deficiency.

#### 2.2.4 Statistical Analysis

Data were analyzed using SPSS 26.0 statistical software. The Mann–Whitney *U* test was used for between‐group comparisons at each screening stage (initial screening and re‐screening). The Wilcoxon signed‐rank test was used for within‐group comparisons between the two screening stages. Quantitative data were expressed as median (minimum–maximum). A two‐tailed *p* < 0.05 was considered statistically significant.

#### 2.2.5 Treatment and Follow‐Up

All children were advised to avoid hunger and prolonged strenuous exercise. Energy supplementation was required when fasting or surgery to prevent disease episodes. Patients under treatment received oral L‐carnitine at a dose of 50–100 mg/(kg d), and blood C0 levels were measured every 2 weeks to 1 month during the initial treatment period. Follow‐up was conducted every 6 months during infancy and annually during childhood, including assessments of blood C0 levels, other acylcarnitine profiles, as well as physical and neurodevelopmental status assessed through routine clinical child health evaluation. Mothers of infants with maternal carnitine deficiency were asymptomatic, and they were informed of the potential health risks and advised to have L‐carnitine supplementation.

## 3 Results

### 3.1 MS/MS and Genetic Testing in NBS

From January 2015 to December 2024, a total of 439,486 newborns were screened using MS/MS. Among them, 1518 cases were identified with C0 < 10 μmol/L, yielding a positive rate of 0.35%. Of these, 1517 newborns were recalled for re‐screening, with a re‐screening recall rate of 99.93%. Among the 1517 recalled newborns, 154 tested positive with persistently low C0 levels at the re‐screening stage. A total of 152 newborns proceeded to the diagnostic stage, with a diagnostic evaluation compliance rate of 98.70%. Ultimately, 20 newborns with a clinically relevant diagnosis were identified, giving a diagnostic yield of 13.16% (20/152) at the diagnostic stage. Among these, 17 newborns were diagnosed with PCD, of whom 15 carried two *SLC22A5* gene variants, with their diagnoses molecularly confirmed with biochemical results. The remaining two carried one variant each and were clinically inferred based on persistent biochemical abnormalities. The screening‐detected prevalence of PCD among newborns in Yancheng was 1:25,852. Additionally, three cases presented with persistently low C0 levels secondary to maternal carnitine deficiency. The remaining 132 cases had C0 levels above the cutoff value at the diagnostic stage. None of them carried two variants, 23 carried one variant, and no variants were detected in the rest.

Based on the final diagnostic results above, 152 newborns who completed diagnostic evaluation were categorized into three groups: the PCD group, the maternal carnitine deficiency‐related group, and the false‐positive group (Figure 1). In the PCD group, the initial screening C0 level was 7.33 (3.87–9.81) μmol/L, and the re‐screening C0 level was 4.98 (2.90–9.84) μmol/L. In the maternal carnitine deficiency‐related group, the initial screening C0 level was 4.33 (3.99–5.47) μmol/L, which increased to 4.98 (4.80–5.93) μmol/L at the re‐screening stage. In the false‐positive group, the initial screening C0 level was 8.45 (2.13–9.80) μmol/L, which decreased to 8.22 (4.14–9.96) μmol/L at the re‐screening stage (Table 1, Figure 2).

**FIGURE 1:**
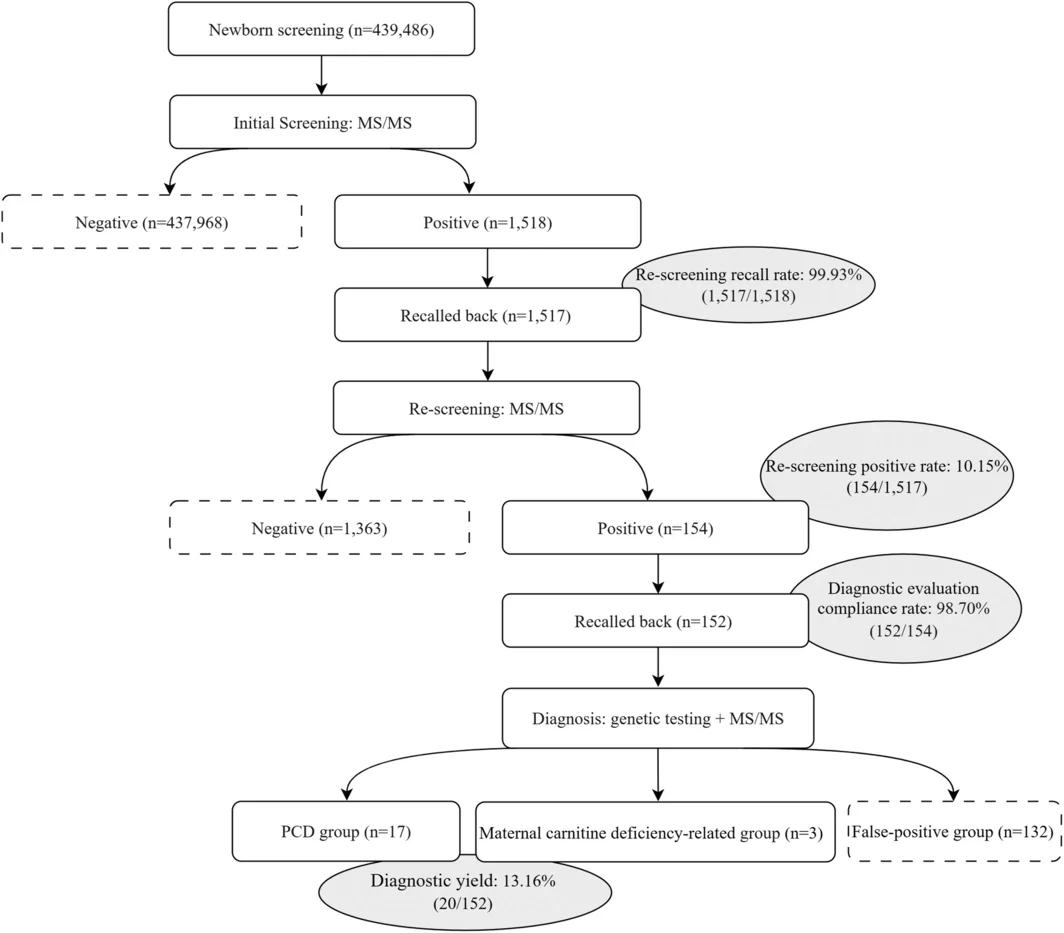
Flowchart and classification of NBS. The PCD group (*n* = 17) included 15 cases molecularly confirmed with biochemical results and two cases clinically inferred. The maternal carnitine deficiency‐related group (*n* = 3) included three newborns presenting with persistently low C0 levels secondary to maternal carnitine deficiency. The false‐positive group (*n* = 132) comprised newborns whose C0 levels returned above the cutoff value at the diagnostic stage.

**TABLE 1:** C0 levels of newborns in each group at different screening stages.

Group	Samples (*n*)	Initial screening C0 levels (μmol/L)	Re‐screening C0 levels (μmol/L)
PCD group	17	7.33 (3.87–9.81)	4.98 (2.90–9.84)
Maternal carnitine deficiency‐related group	3	4.33 (3.99–5.47)	4.98 (4.80–5.93)
False‐positive group	132	8.45 (2.13–9.80)	8.22 (4.14–9.96)

*Note:* Data are presented as median (min–max).

**FIGURE 2:**
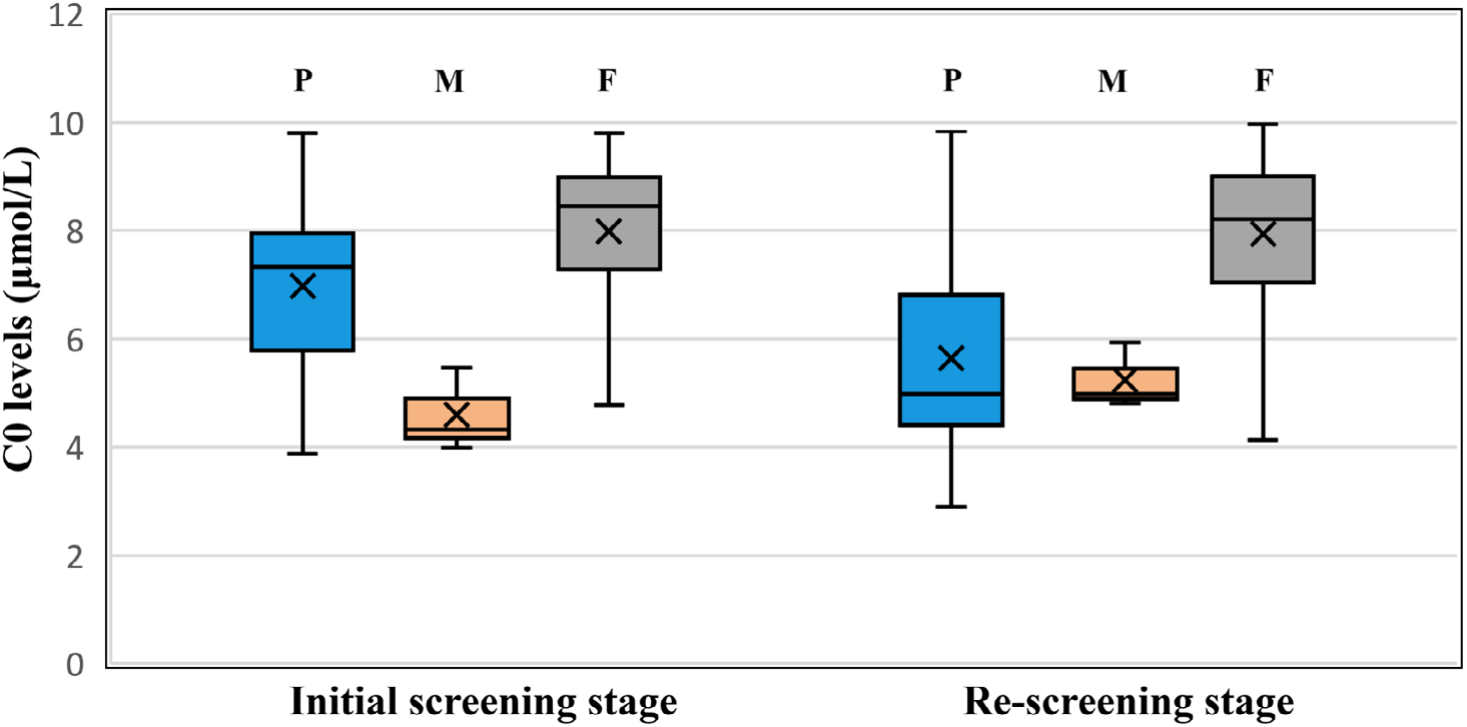
C0 levels at the initial‐screening and re‐screening stages among newborns who completed diagnostic evaluation. Boxes represent the 25th and 75th percentiles, whiskers extend to the observed minimum and maximum values. The horizontal line inside each box denotes the median, and the × symbol denotes the mean. F: False‐positive group (*n* = 132); M: Maternal carnitine deficiency‐related group (*n* = 3); P: PCD group (*n* = 17).

During both the initial and re‐screening stages, the PCD group had significantly lower C0 levels than the false‐positive group. The Mann–Whitney *U* test revealed significant differences between the two groups (initial screening stage: *U* = 650.000, *p* = 0.005) (re‐screening stage: *U* = 433.000, *p* < 0.001). After re‐screening, C0 levels decreased in both groups. In the PCD group, the C0 level at re‐screening was significantly lower than that at the initial screening stage (Wilcoxon signed‐rank test, *p* = 0.007). In the false‐positive group, the C0 level at re‐screening was slightly lower than that at the initial screening stage, but the difference was not statistically significant (Wilcoxon signed‐rank test, *p* = 0.594). In contrast to the above two groups, the maternal carnitine deficiency‐related group showed a higher C0 level at the re‐screening stage. However, due to the small sample size, no hypothesis testing was performed.

### 3.2 Clinical Manifestations, Biochemical and Molecular Genetic Characteristics of Patients

In the 17 PCD newborns, 32 variants involving 10 distinct variant sites were identified, all of which were located in exonic regions (Table 2). Missense variants predominated among the detected variants, with c.1400C>G (p.S467C) being the most frequent (13/32, 40.63%), suggesting it was the hotspot variant in this region.

**TABLE 2:** Detection of *SLC22A5* gene variant sites in 17 PCD newborns.

Variant	Position	Amino acid change	Type	Pathogenicity	Number of detected variants (Frequency)
c.51C>G	Exon 1	p.F17L	Missense Variant	P	7 (21.88%)
c.338G>A	Exon 1	p.C113Y	Missense Variant	P	1 (3.13%)
c.428C>T	Exon 2	p.P143L	Missense Variant	P	3 (9.38%)
c.745_748del	Exon 4	p.F249Lfs*14	Frameshift Variant	LP	1 (3.13%)
c.760C>T	Exon 4	p.R254*	Nonsense Variant	P	1 (3.13%)
c.1319C>T	Exon 8	p.T440M	Missense Variant	P	2 (6.25%)
c.1340A>G	Exon 8	p.Y447C	Missense Variant	P	1 (3.13%)
c.1400C>G	Exon 8	p.S467C	Missense Variant	P	13 (40.63%)
c.1433C>T	Exon 8	p.P478L	Missense Variant	P	1 (3.13%)
c.1445A>G	Exon 8	p.Y482C	Missense Variant	LP	2 (6.25%)

*Note:* Reference genome: GRCh37/hg19, Transcript number: NM_003060.3. Variant pathogenicity was classified according to the ACMG guidelines, LP: likely pathogenic, P: pathogenic.

Among the 17 PCD cases, 15 carried two variants of the *SLC22A5* gene, including four cases with homozygous *SLC22A5* variants and 11 cases with two heterozygous variants (Table 3). Cases 15, 16 and 17 carried the same homozygous variant c.1400C>G (p.S467C). Cases 4, 12 and 14 shared the same two heterozygous variants c.51C>G (p.F17L) and c.1400C>G (p.S467C). The remaining cases had distinct genotypes. Cases 3 and 11 carried only one heterozygous variant site each, but they had C0 levels < 10 μmol/L in three consecutive measurements, with both mothers having normal carnitine levels. The two newborns had no underlying diseases, no history of special medication, no elevation of other acylcarnitines, and normal urinary organic acids. Therefore, secondary carnitine deficiency was excluded, and they were clinically inferred to have PCD. Cases 18, 19, and 20 had C0 levels < 10 μmol/L at the diagnostic stage, with maternal C0 levels of 5.10, 3.62, and 2.57 μmol/L, respectively. They were therefore diagnosed with maternal carnitine deficiency. Two of them were heterozygous for c.1400C>G (p.S467C) (Table 3). The mother of Case 18 refused further genetic testing for herself, while the mothers of Cases 19 and 20 were found to carry homozygous variant c.1400C>G (p.S467C) and were diagnosed with PCD.

**TABLE 3:** Biochemical and genetic characteristics of 20 newborns with a clinically relevant diagnosis.

Group	Case	C0 levels (μmol/L)			*SLC22A5* gene		Follow‐Up
		Initial screening stage	Re‐screening stage	Diagnostic stage	Variant 1	Variant 2	
PCD group	1	7.33	4.83	3.96	c.51C>G (p.F17L)	c.1445A>G (p.Y482C)	Remained on treatment, asymptomatic
	2	7.86	5.57	6.24	c.1400C>G (p.S467C)	c.1433C>T (p.P478L)	Remained on treatment, asymptomatic
	3	6.89	4.84	4.89	c.1400C>G (p.S467C)	/	Untreated, asymptomatic
	4	7.95	5.10	4.75	c.51C>G (p.F17L)	c.1400C>G (p.S467C)	Lost to follow‐up
	5	6.21	8.31	9.03	c.428C>T (p.P143L)	c.428C>T (p.P143L)	Discontinued treatment, asymptomatic
	6	6.32	4.03	3.33	c.338G>A (p.C113Y)	c.1400C>G (p.S467C)	Remained on treatment, asymptomatic
	7	4.65	4.98	5.20	c.760C>T (p.R254*)	c.1400C>G (p.S467C)	Remained on treatment, poor exercise tolerance
	8	8.57	5.44	4.93	c.428C>T (p.P143L)	c.1319C>T (p.T440M)	Remained on treatment, asymptomatic
	9	5.78	2.90	3.21	c.745_748del (p.F249Lfs*14)	c.1319C>T (p.T440M)	Remained on treatment, asymptomatic
	10	5.66	3.08	1.89	c.51C>G (p.F17L)	c.1340A>G (p.Y447C)	Untreated, asymptomatic
	11	7.55	6.81	8.46	c.51C>G (p.F17L)	/	Untreated, asymptomatic
	12	8.47	4.63	5.47	c.51C>G (p.F17L)	c.1400C>G (p.S467C)	Remained on treatment, asymptomatic
	13	5.52	4.41	4.69	c.51C>G (p.F17L)	c.1445A>G (p.Y482C)	Remained on treatment, asymptomatic
	14	3.87	4.40	6.03	c.51C>G (p.F17L)	c.1400C>G (p.S467C)	Remained on treatment, asymptomatic
	15	8.34	7.48	3.56	c.1400C>G (p.S467C)	c.1400C>G (p.S467C)	Remained on treatment, asymptomatic
	16	9.81	9.35	9.26	c.1400C>G (p.S467C)	c.1400C>G (p.S467C)	Untreated, asymptomatic
	17	7.92	9.84	7.47	c.1400C>G (p.S467C)	c.1400C>G (p.S467C)	Remained on treatment, asymptomatic
Maternal carnitine deficiency‐related group	18	5.47	4.80	5.70	/	/	Formula feeding, asymptomatic
	19	3.99	5.93	6.07	c.1400C>G (p.S467C)	/	Discontinued treatment, asymptomatic
	20	4.33	4.98	6.41	c.1400C>G (p.S467C)	/	Discontinued treatment, asymptomatic

*Note:* /: not detected.

At the diagnostic stage, none of the 20 newborns with a clinically relevant diagnosis presented with obvious clinical manifestations. All 17 PCD newborns followed medical advice to reduce risks of infection, hunger, and strenuous exercise, and were advised to seek timely medical attention if related symptoms occurred. The treated newborns received L‐carnitine at a dose of 50–100 mg/(kg d). Case 4 was transferred to a superior hospital for treatment at the guardian's request and was lost to follow‐up. Case 5 discontinued medication after 1 year of age due to adverse reactions of vomiting and abdominal pain. For three newborns with maternal carnitine deficiency, case 18 was switched to formula feeding, and cases 19 and 20 discontinued medication at around 1 month of age. The subsequent C0 levels of these maternal cases returned to normal.

Due to age differences among these cases, the follow‐up period ranged from 18 months to 10 years. During the follow‐up period, none of the cases developed acute metabolic decompensation. With the exception of Case 7, who had poor exercise tolerance, all other patients had normal intellectual, motor, and growth development assessments. The mothers of the maternal carnitine deficiency cases received standard treatment and showed no related abnormalities.

### 3.3 Prenatal Diagnosis for the Recurrence‐Risk Family

Sanger sequencing confirmed that the pregnant woman carried a heterozygous variant of c.865C>T (p.R289*) in the *SLC22A5* gene, with no c.1400C>G (p.S467C) variant was detected. Her husband carries a heterozygous variant of c.1400C>G (p.S467C) in the *SLC22A5* gene, with no c.865C>T (p.R289*) variant was detected. The two variants of the proband were inherited from the mother and father, respectively. The sampled amniotic fluid contained the maternal c.865C>T (p.R289*) heterozygous variant, but no c.1400C>G (p.S467C) variant was found (Figure 3). Following genetic counseling, the couple decided to continue the pregnancy. The postnatal NBS result revealed that the infant's C0 level was within the normal range, and no abnormalities were discovered during subsequent follow‐up.

**FIGURE 3:**
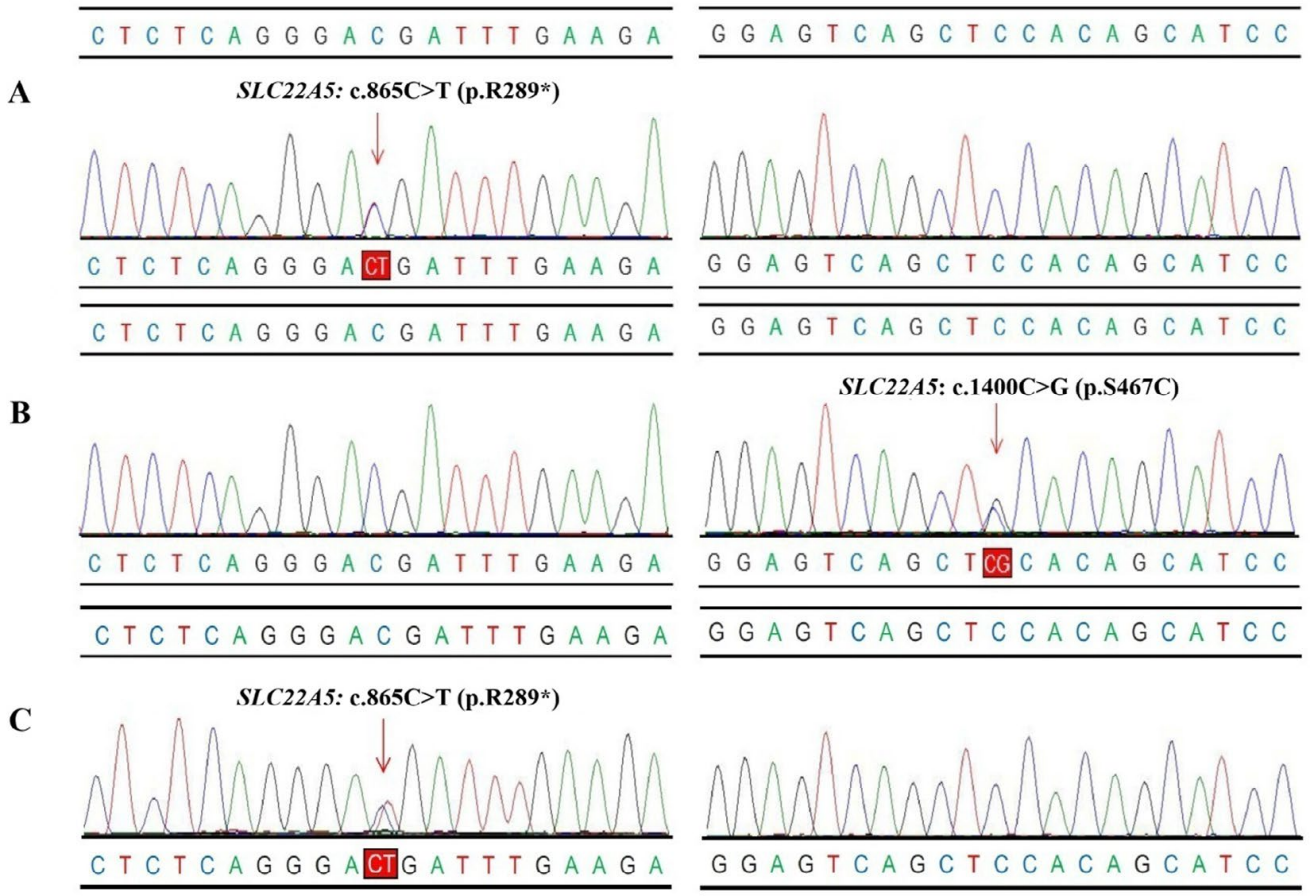
Sanger Sequencing of *SLC22A5* of the Prenatal Diagnosis Family. (A) The pregnant woman had a heterozygous variant of c.865C>T (p.R289*). (B) The husband had a heterozygous variant of c.1400C>G (p.S467C). (C) The fetal amniotic fluid had a maternal heterozygous variant of c.865C>T (p.R289*).

## 4 Discussion

The OCTN2 protein encoded by the *SLC22A5* gene transports carnitine into cells across the plasma membrane and reabsorbs carnitine in the renal proximal tubules to maintain intracellular carnitine concentrations. Reduced intracellular carnitine availability impairs β‐oxidation of long‐chain fatty acids, giving rise to the various clinical manifestations of PCD (Kheirandish et al. 2024; Jolfayi et al. 2024). MS/MS measurement of C0 levels is now widely regarded as the most reliable and rapid method for PCD screening and diagnosis. In China, based on expert consensus recommendations, the blood C0 cutoff value is uniformly set at 10 μmol/L for both newborn and non‐newborn screening or diagnosis. To reduce unnecessary recalls and the waste of medical resources, some countries and regions have set the lower cutoff value at 4–7.3 μmol/L (Alshehri et al. 2024; Crefcoeur et al. 2023; Ambrose et al. 2022; Chan et al. 2024; Jia et al. 2021). In our study, however, the initial screening C0 level in the PCD group was 7.33 (3.87–9.81) μmol/L, and 8 out of 17 PCD cases had initial C0 values ranging from 7.5 to 10 μmol/L. Therefore, we consider that adopting an excessively low cutoff value during initial screening may lead to missed screening and subsequent false‐negative results.

Most carnitine in the human body is obtained from diet, with a small portion synthesized in the liver and kidneys. C0 is freely transported from the mother to the fetus through the placenta during pregnancy, and neonatal carnitine levels are also strongly influenced by maternal factors (Gallant et al. 2017; Lin et al. 2024). Newborn PCD screening by measuring C0 levels inevitably faces a high false‐positive rate (Koleske et al. 2022; Lefèvre et al. 2023). Physiological carnitine decline during pregnancy, premature sample collection after birth, insufficient intake from exclusive breastfeeding, prematurity, low birth weight, and cesarean section are all factors that can lead to false‐positive results (Chang et al. 2022; Turturo et al. 2026). In this study, among the 152 newborns recalled for diagnostic evaluation, only 20 were with a clinically relevant diagnosis giving a diagnostic yield of 13.16%. This value was lower than that reported in previous studies from other regions of China, which may be related to their setting of the C0 cutoff at 9.50 μmol/L (Yang et al. 2021). Due to the protective effect of normal mothers on PCD newborns, their blood C0 levels do not reach the minimum value by day 3 after birth (Ji et al. 2023). In the present study, the C0 levels in the PCD group decreased significantly during re‐screening, which is consistent with the above observations. Although the C0 levels in the PCD group were significantly lower than those in the false‐positive group during both the initial and re‐screening stages, there was range overlap among the three groups. Therefore, the diagnosis and classification of PCD cannot rely solely on C0 measurements from the two screening stages, and *SLC22A5* variant testing serves as one of the major diagnostic criteria for PCD. Compared with fibroblast carnitine transporter activity assays, genetic testing features a shorter turnaround time and lower invasiveness (Zhi et al. 2025; Fecarotta et al. 2025).

The *SLC22A5* gene consists of 10 exons and 9 introns. Missense variants, nonsense variants, splice site variants, and deletions of this gene can all lead to OCTN2 functional defects (Jolfayi et al. 2024). The dbSNP database (accessed May 31, 2026) contained 11,870 variants in the *SLC22A5* gene, of which 256 are classified as pathogenic/likely pathogenic (P/LP), with missense variants being the predominant type (Galluccio et al. 2024). Data from gnomAD v4.1.0 revealed that most P/LP variants are located in exons, with exon 1 being the most frequently affected. In this study, a total of 10 distinct variant sites were detected in the 17 PCD patients, including 8 missense variants, 5 of which were located in exon 8. Hotspot variants of the *SLC22A5* gene vary across ethnicities and regions, and the identification of these hotspots facilitates more efficient molecular probe development (Tan et al. 2021). The common variant in Italy is c.505C>T, while that in the United States is c.136C>T (Hu et al. 2025). c.1400C>G ranks among the most frequent variants in Japan and has not been reported in non‐East Asian populations (Ji et al. 2023). Large‐scale studies in the Chinese population have identified c.1400C>G, c.51C>G, and c.760C>T as common variants, though the most frequent variant varies by region (Zhou et al. 2024; Lin et al. 2020). In our region, among the PCD newborns diagnosed through MS/MS screening, c.1400C>G had the highest prevalence (13/32, 40.63%), markedly higher than the second one c.51C>G (7/32, 21.88%), while the frequency of c.760C>T was very low (1/32, 3.13%). This suggests that c.1400C>G is the most prevalent variant in this region.

In the PCD group, Cases 3 and 11 carried only one pathogenic variant. However, after exclusion of secondary carnitine deficiency, their C0 levels remained persistently below the cutoff value across three consecutive measurements, supporting a PCD diagnosis based on clinical inference. Such cases that are biochemically diagnosed without molecular confirmation are not uncommon. Hu et al. reported that among 44 PCD newborns diagnosed at their center, two cases had no *SLC22A5* variants detected (Hu et al. 2025). This may arise because the next‐generation sequencing technology used in our study only detects point variants and small insertions/deletions (indels ≤ 10 bp) within the coding sequences of the target gene and the flanking ±10 bp intronic regions. This approach cannot identify potential pathogenic variants located in gene regulatory regions and deep intronic regions, nor can it detect gene methylation modifications. Frigeni et al. sequenced the 10 exons and flanking regions of the *SLC22A5* gene in subjects with impaired fibroblast carnitine transport and found that pathogenic variants were present in merely 84% of alleles (Frigeni et al. 2017). Ferdinandusse et al. performed genetic sequencing on 236 individuals suspected of PCD and found that only 56.4% of them carried biallelic pathogenic variants (Ferdinandusse et al. 2019). This explains why we arranged genetic testing for re‐screening positive newborns while still requiring repeated biochemical tests. This effectively prevents missed diagnoses caused by the technical limitations of genetic testing.

This indicates that the diagnosis of PCD cannot overly rely on genetic testing results but must be made through comprehensive evaluation combined with biochemical indicators. Some studies have reported that individuals with nonsense or frameshift variants tend to have lower C0 levels than those with missense variants, and variants closer to the C‐terminal domain correlate with lower C0 levels (Chang et al. 2022; Hu et al. 2025). Due to the small number of confirmed PCD patients in this study, correlation analysis between variant sites or types and C0 levels or phenotypes was not performed. Meanwhile, other researchers still hold the view that the genotype–phenotype correlation of PCD remains unclear. That is, the same variant may lead to distinct phenotypes, while the same phenotype may result from different variants (Zhou et al. 2024). We provide the C0 levels at the initial, re‐screening, and diagnostic stages, along with genetic testing results and other clinical data of the newborns with a clinically relevant diagnosis for further research and analysis by other scholars. For the PCD recurrence‐risk family, we verified that the pathogenic variants of the proband were inherited from each parent. Prenatal diagnosis revealed that the fetus carried only the maternal variant. Given the autosomal recessive inheritance pattern and the absence of abnormal phenotypes in both parents, the fetus had an extremely low probability of having PCD. After birth, the infant's blood acylcarnitine profile showed no obvious abnormalities, and normal growth and motor development were observed during follow‐up. This indicates that *SLC22A5* gene testing provided an effective basis for prenatal diagnosis in this family. Since this observation is based on a single recurrence‐risk family with a previously affected proband, it should be regarded as a case illustration of the feasibility of prenatal diagnosis in such families rather than as general evidence of its clinical utility. The principal value of this approach lies in providing informed reproductive counseling for recurrence‐risk families, especially given that some patients with PCD may remain asymptomatic while others may decline treatment.

PCD patients generally have unremarkable birth conditions, with the average age of onset around 2 years, mainly presenting with hepatomegaly, muscle weakness, cardiomyopathy, and cardiac dysfunction. Although some adult patients are asymptomatic, they still have a risk of sudden cardiac death (Longo et al. 2016). Multiple cases of sudden death among young adults in the Faroe Islands have been attributed to untreated PCD (Abrahamsen et al. 2023). PCD is often triggered by stress states such as fasting, prolonged strenuous exercise, infection, and surgery. Early diagnosis and oral L‐carnitine supplementation before the occurrence of irreversible damage contribute to a favorable prognosis (Jain et al. 2022). In this study, 17 PCD newborns and three mothers with low C0 levels were provided with presymptomatic diagnosis and treatment plans owing to participation in NBS. Four of the 17 PCD newborns chose not to take medication initiatively, and promised to avoid trigger factors and seek timely medical attention if suspected symptoms of PCD appeared. The three maternal carnitine deficiency‐related newborns all had their C0 levels return to normal after medication or adjustment of feeding methods, and no related symptoms occurred during subsequent follow‐up. This study also has several limitations: (1) The number of confirmed PCD patients was small, which precluded the exploration of correlations between variant sites or types and C0 levels or phenotypes. (2) One patient was lost to follow‐up. (3) The prenatal diagnosis findings, being based on one family, should likewise be interpreted as a case illustration.

## 5 Conclusion

Substantial individual differences exist in C0 values detected by MS/MS during NBS for PCD and preclude diagnosis and classification based solely on numerical values. Combining MS/MS with genetic testing enables early diagnosis of neonatal PCD. The screening‐detected prevalence of PCD in Yancheng, China, is 1:25,852. The *SLC22A5* gene variants in this region primarily involve missense variants, with c.1400C>G (p.S467C) identified as the hotspot variant. Findings from one affected family further support the feasibility of *SLC22A5* genetic testing for prenatal diagnosis and genetic counseling in recurrence‐risk families.

## Author Contributions

**Huilin Sun:** writing – original draft, formal analysis, visualization, funding acquisition. **Jianli Zheng:** writing – review and editing, investigation. **Chong Zhou:** investigation, data curation, writing – review and editing. **Haihong Chen:** data curation, investigation. **Lu Zhang:** visualization, formal analysis. **Xiaorui Li:** data curation, validation. **Daocheng Bao:** conceptualization, writing – review and editing, supervision, methodology. **Fangfang Yang:** data curation, writing – review and editing, investigation, supervision, validation. **Jianbing Liu:** writing – review and editing, validation, methodology, conceptualization, investigation.

## Funding

This work was supported by the Yancheng Science and Technology Bureau (YCBE202465), Jiangsu Vocational College of Medicine Collaborative Innovation Research Project between School and Campus (202491309), and Jiangsu Province Youth Science and Technology Talent Support Project (JSTJ‐2024‐580).

## Ethics Statement

This study was approved by the ethics committee of Yancheng Maternal and Child Health Care Hospital Affiliated to Yangzhou University (2025‐LW‐012), Yancheng, China, and complied with the Helsinki Declaration.

## Consent

All participants or guardians signed informed consent forms.

## Conflicts of Interest

The authors declare no conflicts of interest.

## Data Availability

The datasets supporting the main findings of this study are included in the article. Due to laboratory confidentiality policies, ethical requirements, and patient informed consent, we are unable to submit raw sequencing data to publicly available databases. Additional information can be obtained from the corresponding author upon reasonable request.
